# Prevalence and molecular characterization of pyrazinamide resistance among multidrug-resistant *Mycobacterium tuberculosis* isolates from Southern China

**DOI:** 10.1186/s12879-017-2761-6

**Published:** 2017-11-06

**Authors:** Yu Pang, Damian Zhu, Huiwen Zheng, Jing Shen, Yan Hu, Jie Liu, Yanlin Zhao

**Affiliations:** 1 National Clinical Laboratory on Tuberculosis, Beijing Key Laboratory on Drug-Resistant Tuberculosis Research, Beijing Chest Hospital, Capital Medical University, Beijing Tuberculosis and Thoracic Tumor Institute, Beijing, China; 2 Clinical Laboratory, Chongqing Tuberculosis Control Institute, No. 71, Longteng Street, Jiulongpo District, Chongqing, 400050 People’s Republic of China; 3 National Center for Tuberculosis Control and Prevention, Chinese Center for Disease Control and Prevention, No. 155, Chang Bai Road, Changping District, Beijing, 102206 People’s Republic of China

## Abstract

**Background:**

Pyrazinamide (PZA) plays a unique role in the treatment for multidrug-resistant tuberculosis (MDR-TB) in both first- and second-line regimens. The aim of this study was to investigate the prevalence and molecular characterization of PZA resistance among MDR-TB isolates collected in Chongqing municipality.

**Methods:**

A total of 133 MDR-TB isolates were collected from the smear-positive tuberculosis patients who were registered at local TB dispensaries of Chongqing. PZA susceptibility testing was determined with a Bactec MGIT 960 system. In addition, the genes conferring for PZA resistance were screened by DNA sequencing.

**Results:**

Of these 133 MDR-TB isolates, 83 (62.4%) were determined as PZA-resistant by MGIT 960. In addition, streptomycin- (83.1% vs. 56.0%, *P* < 0.01), ofloxacin- (51.8% vs. 18.0%, *P* < 0.01), kanamycin- (22.9% vs. 2.0%, *P* < 0.01), amikacin- (18.1% vs. 2.0%, *P* = 0.01), capromycin-resistance (12.0% vs. 2.0%, *P* = 0.05), were more frequently observed among PZA-resistant isolates compared with PZA-susceptible isolates. Sequence analysis revealed that 73 out of 83 (88.0%) MDR strains harbored a mutation located in the *pncA* gene, including 55 (75.3%, 55/73) of single nucleotide substitutions and 18 (24.7%, 18/73) of frameshift mutation, while no genetic mutation associated with PZA resistance was found in the *rpsA* gene. The *pncA* expression of strains harboring substitution from A to G at position −11 in the promoter region of *pncA* was significantly lower than that of H37Rv (*P* < 0.01).

**Conclusions:**

In conclusion, our data have demonstrated that the analysis of the *pncA* gene rather than *rpsA* gene provides rapid and accurate information regarding PZA susceptibility for MDR-TB isolates in Chongqing. In addition, loss of *pncA* expression caused by promoter mutation confers PZA resistance in MDR-TB isolates.

## Background

Multidrug resistant tuberculosis (MDR-TB) is a major concern hampering global tuberculosis control efforts [1, 2]. According to new estimates from World Health Organization (WHO), there were around 0.48 million new cases of MDR-TB cases, and approximately 0.19 million deaths from MDR-TB worldwide in 2014 (WHO, 2015). Only behind India, China has the second burden of MDR-TB globally, with 52, 000 prevalent MDR-TB cases annually (WHO, 2015). A recent national survey of drug-resistant tuberculosis in China revealed that 5.7% of new TB cases and 25.6% of previously treated cases were MDR-TB, respectively, which were higher than the global average rates [3]. Given its high rate of treatment failure, the epidemic of MDR-TB constitutes a serious public health problem in China [3, 4].

Pyrazinamide (PZA) is one of cornerstone first-line anti-tuberculosis agents that is also commonly used as essential component in the therapeutic treatment of MDR-TB [5, 6]. As the prodrug, PZA requires conversion into its active form pyrazinoic acid (POA) by the enzyme pyrazinamidase (PZase). PZase is encoded by the 561-nucleotide *pncA* gene. Loss of PZase activity cause by genetic mutations in the *pncA* gene is the main mechanism of resistance to PZA in *M. tuberculosis* [6]. Several recent literatures have reported that several PZA-resistant *M. tuberculosis* isolates harbored the mutations in the promoter of *pncA* gene, indicating these alternations may result in PZA resistance by influencing the expression of *pncA* [7–9]. In addition to *pncA*, another gene named *rpsA*, which encodes the 30S ribosomal protein S1, has been demonstrated to confer PZA resistance by structural alternation of POA binding [10]. To date, the contribution of the *rpsA* mutations conferring PZA resistance is controversial, which requires more experimental evidences to elucidate the role of *rpsA* as a PZA resistance mechanism [8].

Chongqing is the largest municipality in the southwestern China [11]. Due to the underdeveloped setting, Chongqing is considered as a hotspot for both TB and MDR-TB in China [12]. However, limited data is available for the prevalence and molecular characteristics of PZA resistance in *M. tuberculosis* isolates, especially MDR-TB. In this study, our main goal was to investigate the prevalence of PZA resistance among MDR-TB isolates collected in Chongqing municipality. We also sought to analyze the mutant profiles of MDR-TB isolates conferring PZA resistance in this area.

## Methods

### Patient enrollment

All smear-positive tuberculosis patients who were registered at local TB dispensaries between November 2014 and February 2016 were enrolled in this study. Information was obtained from patient’s medical record. Two sputum samples were obtained from each smear-positive patient for culturing in the county-level laboratories. After 4–8 weeks of incubation, cultures with growing colonies on Löwenstein-Jensen (L-J) medium were sent to the Chongqing Tuberculosis Control Institute for further drug susceptibility testing. Re-treated cases were defined as patients having previously received more than one month of anti-TB treatment.

### Drug susceptibility testing

Drug susceptibility testing (DST) was performed with the proportional method recommended by WHO [3, 13]. The concentrations of drugs in L-J medium were as follows: isoniazid (INH), 0.2 μg/ml; rifampicin (RIF), 40 μg/ml; ethambutol (EMB), 2 μg/ml; streptomycin (SM), 4 μg/ml; ofloxacin (OFLX), 2 μg/ml; kanamycin (KAN), 30 μg/ml; amikacin (AMK), 30 μg/ml; capromycin (CAP), 40 μg/ml; and protionamide (PTO), 40 μg/ml and p-aminosalicylic acid (PAS) 1 μg/ml [14]. A strain was declared resistant to an antimicrobial agent when the growth rate exceeded 1% compared with the control. In addition, PZA susceptibility testing was determined with a Bactec MGIT 960 system according to the manufacturer’s instructions. The critical concentration of PZA in the liquid medium was 100 μg/ml [8]. The MDR-TB strains were defined as those resistant to both isoniazid and rifampicin. In addition, XDR-TB was defined as MDR-TB resistant to any member of the quinolone family and at least one of the remaining second-line anti-TB injectable drugs [15].

### DNA extraction and amplification

Genomic DNA was extracted from freshly cultured bacteria as previously described [16]. Briefly, the harvested bacteria from the surface of L-J medium were suspended in 500 μl Tris-EDTA (TE) buffer and heated in a 95 °C water bath for 30 min. The crude DNA was used as template for amplification. The fragments of *pncA* and *rpsA* were amplified with the following primers: *pncA*-F 5′-AACAGTTCATCCCGGTTC-3′ and *pncA*-R 5′-GCGTCATGGACCCTATATC-3′; *rpsA*-F 5′-CGGAGCAACCCAACAATA-3′ and *rpsA*-R 5′-GTGGACAGCAACGACTTC-3′, respectively [17]. The 50 μL PCR mixture was prepared as follows: 25 μl 2 × GoldStar MasterMix (CWBio, Beijing, China), 5 μL of DNA template, and 0.2 μM of each primer set. PCR parameters for amplification were 5 min at 94 °C followed by 35 cycles of 94 °C for 1 min, 58 °C for 1 min, 72 °C for 1 min and a final extension of 72 °C for 5 min. PCR products were sent to Tsingke company for sequencing. All sequence data were aligned with *pncA* and *rpsA* of reference strain H37Rv (ATCC) using BioEdit (version 7.1.3.0) software.

### Quantitative reverse transcription PCR (QRT-PCR)

Bacteria were harvested from L-J medium after inoculation for 4 weeks. The total RNA was isolated following a standard Trizol RNA extraction protocol supplied by Invitrogen (Invitrogen, Life Technologies, USA) [18]. Followed by treatment with DNaseI (Invitrogen, Life Technologies, USA), the reverse transcription was carried out using SuperScriptIII RT kit (Invitrogen, Life Technologies, USA). The relative expression level of *pncA* gene was detected by QRT-PCR in a 20 μl system containing 10 μL of 2 × UltraSYBR Mixture (CWBio, Beijing, China), 2 μL of cDNA template, and 0.2 μM of each primer set (pncA-QF 5′-GAAGCGGCGGACTACCATC-3′ and pncA-QR 5′-AGTGGCGTGCCGTTCTCG-3′). PolyA was set as the internal control in respective PCR experiments [18].

### Data analysis

Chi square test was used to evaluate the associations among multiple categorical variables, and the statistical results were summarized with odds ratios (ORs) with 95% confidence intervals (CIs). All calculations were performed in SPSS 13.0 (SPSS Inc., USA). Differences with a *P* value less than 0.05 were declared statistically significant.

## Results

### Demographic characteristics and drug susceptibility profiles

A total of 133 (10.8%) of 1236 clinical isolates were identified as MDR-TB, including 38 (28.6%) pre-XDR and 17 (12.8%) XDR. Overall, 80 (60.2%) strains were isolated from male patients and 53 (39.8%) from female patients. The average age of the patients was 46.6 years (range 19–76 years). In addition, 36.8% of isolates were from new cases, and 63.2% from re-treated cases. Out of the 133 MDR isolates tested, 97 (72.9%) were resistant to SM, 40 (30.1%) to EMB, 52 (39.1%) to OFLX, 20 (15.0%) to KAN, 16 (12.0%) to AMK, 11 (8.3%) to CAP and 15 (11.3%) to PAS (Table 1).

**Table 1:** Risk factor associate with PZA resistance among 133 MDR^a^ isolates

Characteristics	No. (%) of isolates (*n* = 133)	No. (%) of isolates		OR (95% CI)	*P* value
		PZA^R^ (*n* = 83)	PZA^S^ (*n* = 50)		
Sex					
Male	80 (60.2)	47 (56.6)	33 (66.0)	0.67 (0.33–1.39)	0.29
Female	53 (39.8)	36 (43.4)	17 (34.0)	1.0 (Ref.)	–
Age group					
< 30	31 (23.3)	18 (21.7)	13 (26.0)	1.0 (Ref.)	–
30–59	80 (60.2)	55 (66.3)	25 (50.0)	1.59 (0.68–3.74)	0.29
≥ 60	22 (16.5)	10 (12.0)	12 (24.0)	0.60 (0.20–1.81)	0.36
Treatment history					
New case	49 (36.8)	22 (26.5)	27 (54.0)	1.00 (Ref.)	–
Re-treated	84 (63.2)	61 (73.5)	23 (46.0)	3.26 (1.55–6.82)	<0.01
Resistance to:					
SM	97 (72.9)	69 (83.1)	28 (56.0)	3.87 (1.74–8.63)	<0.01
EMB	40 (30.1)	26 (31.3)	14 (28.0)	1.17 (0.54–2.54)	0.69
OFLX	52 (39.1)	43 (51.8)	9 (18.0)	4.89 (2.11–11.35)	<0.01
KAN	20 (15.0)	19 (22.9)	1 (2.0)	14.58 (1.88–112.44)	<0.01
AMK	16 (12.0)	15 (18.1)	1 (2.0)	10.81 (1.38–84.58)	0.01
CAP	11 (8.3)	10 (12.0)	1 (2.0)	6.71 (0.83–54.12)	0.05
PAS	15 (11.3)	13 (15.7)	2 (4.0)	4.46 (0.96–20.65)	0.04
Pre-XDR^b^	38 (28.6)	30 (36.1)	8 (16.0)	2.97 (1.23–7.16)	0.02
XDR^c^	17 (12.8)	16 (19.3)	1 (2.0)	11.70 (1.50–91.22)	<0.01

^a^MDR is defined as *Mycobacterium tuberculosis* strain resistant to at least isoniazid and rifampin

^b^Pre-XDR is defined as MDR strain additionally resistant to either ofloxacin or kanamycin, but not both

^c^XDR is defined as *Mycobacterium tuberculosis* strain resistant to isoniazid, rifampin, ofloxacin and kanamycin

### Factors associated with PZA resistance

As shown in Table 1, 83 (62.4%) isolates were determined as PZA-resistant by MGIT, while the other 50 (37.6%) were susceptible to PZA. In regard to the distribution of MDR-TB cases treatment history, the percentage of re-treated MDR-TB patients in the PZA-resistant group was significantly higher than in the PZA-susceptible group (odd ratio (OR) [95% confidence interval (CI): 3.26[1.55–6.82], *P* < 0.01). In contrast, our data revealed no statistically significant difference between the PZA-resistant and PZA-susceptible group in gender and age (*P* > 0.05).

We further analyzed the resistance profiles of other drugs among PZA-resistant and PZA-susceptible *M. tuberculosis* strains. Statistical analysis revealed that SM- (83.1% vs. 56.0%, *P* < 0.01), OFLX- (51.8% vs. 18.0%, *P* < 0.01), KAN- (22.9% vs. 2.0%, *P* < 0.01), AMK- (18.1% vs. 2.0%, *P* = 0.01), CAP-resistance (12.0% vs. 2.0%, *P* = 0.05), pre-XDR (36.1% vs. 16.0%, *P* < 0.02) and XDR (19.3% vs. 2.0%, *P* < 0.01) were more frequently observed among PZA-resistant isolates compared with PZA-susceptible isolates, while no difference was identified between EMB-resistant and EMB-susceptible strains (31.3% vs. 28.0%, *P* = 0.69) (Table 1).

### Mutations in the *pncA* and *rpsA* gene

A total of 73 out of 83 (88.0%) MDR strains harbored a mutation located in the *pncA* gene, including 55 (75.3%, 55/73) of single nucleotide substitutions and 18 (24.7%, 18/73) of frameshift mutation. As summarized in Table 2, we observed great mutant diversity in *pncA* gene, and there were 48 different mutant types conferring PZA resistance among MDR strains in Chongqing. The most prevalent mutation associated with PZA resistance was found in codon 132 of *pncA* (12.3%, 9/73), resulting in the amino aicd substitution of Gly to Asp. As the second affected codon, five isolates had a mutation in codon 62 (6.8%, 5/73). Notably, the third frequent mutation was the nucleotide substitution from A to G at position −11 in the promoter region of *pncA*, accounting for 5.4% of mutant isolates. We also found that 4 PZA-susceptible isolates carried a genetic mutation in *pncA*, including 2 strains in codon 62, one in codon 67 and one in codon 154 (Table 3). In addition, no genetic mutation associated with PZA resistance was found in the *rpsA* gene in this study.

**Table 2:** Mutations of PZA-resistant MDR-TB isolates within *pncA*

Locus	Position of nucleotide	Nucleotide substitution	Position of amino acid	Amino acid substitution	No. of isolates
*pncA*	−11	TAT → TGT	−4	Tyr → Cys	4
	2	ATG → ACG	1	Met → Thr	1
	20	GTC → GGC	7	Val → Gly	2
	24	GAC → GAG	8	Asp → Glu	1
	28	CAG → TAG	10	Gln → Stop	1
	35	GAC → GCC	12	Asp → Ala	2
	37	TTC → GTC	13	Phe → Val	1
	40	TGC → CGC	14	Cys → Arg	2
	40	TGC → CGC	14	Cys → Arg	1
	94	TTC → GTC	32	Phe → Val	1
	123	TAC → TAG	41	Tyr → Stop	1
	146	GAC → GCC	49	Asp → Gly	1
	146	GAC → GCC	49	Asp → Ala	2
	151	CAC → TAC	51	His → Tyr	1
	151	CAC → CGC	51	His → Arg	1
	152	CAC → CCC	51	His → Pro	1
	170	CAC → CGC	57	His → Arg	1
	185	CCG → CTG	62	Pro → Leu	5
	206	CCG → CTG	69	Pro → Leu	1
	213	CAT → CAG	71	His → Gln	1
	226	ACT → CCT	76	Thr → Pro	3
	232	GGC → AGC	78	Gly → Asp	1
	245	CAT → CGT	82	His → Arg	1
	286	AAG → CAG	96	Lys → Gln	1
	307	TAC → CAC	103	Tyr → His	1
	309	TAC → TAG	103	Tyr → Stop	1
	319	GAA → AAA	107	Glu → Lys	1
	395	GGT → GAT	132	Gly → Asp	9
	425	ACG → ATG	142	Thr → Met	1
	437	GCG → GTG	146	Ala → Val	1
	464	GTG → GGG	155	Val → Gly	1
	488	GTG → GCG	163	Val → Ala	1
	515	CTG → CCG	172	Leu → Pro	2
	52	insertion of GC			1
	130	insertion of C			1
	136	deletion of G			2
	139	insertion of CA			1
	232	insertion of C			1
	243	insertion of T			1
	288	insertion of A			1
	341	deletion of ACGCC			1
	342	deletion of GCCAC			2
	376	deletion of GATGAGGTC			1
	392	insertion of G			1
	392	insertion of GG			1
	393	insertion of GGT			1
	408	insertion of CA			1
	408	insertion of A			2
Total					73

**Table 3:** Mutations in PZA-susceptible MDR-TB isolates within *pncA* gene

Locus	Position of nucleotide	Nucleotide substitution	Position of amino acid	Amino acid substitution	No. of isolates
*pncA*	184	CCG → ACG	62	Pro → Thr	1
	185	CCG → CAG	62	Pro → Gln	1
	200	TCG → TGG	67	Ser → Trp	1
	461	AGG → AAG	154	Arg → Lys	1
Total					4

We analyzed the performance of *pncA* mutations for predicting PZA susceptibility. When setting the phenotypic PZA susceptibility as a gold standard, we found that detection of mutations in *pncA* gene exhibited a sensitivity of 88.0% (95% CI, 80.9%–95.0%) and a specificity of 92.0% (95% CI, 84.5%–99.5%) (Table 4).

**Table 4:** Performance of *pncA* mutations for predicting PZA susceptibility

Mutation in *pncA*	PZA susceptibility^a^		Total	Sensitivity (95% CI, %)	Specificity (95% CI, %)	PPV (95% CI, %)	NPV (95% CI, %)
	R	S					
Yes	73	4	77	88.0 (80.9–95.0)	92.0 (84.5–99.5)	94.8 (89.8–99.8)	82.1 (72.1–92.2)
No	10	46	56				
Total	83	50	133				

^a^ *R*, resistant, *S* susceptible, *PPV* positive predictive value, *NPV* negative predictive value, *CI* confidence interval

### Loss of *pncA* expression due to promoter mutation

**Fig. 1:**
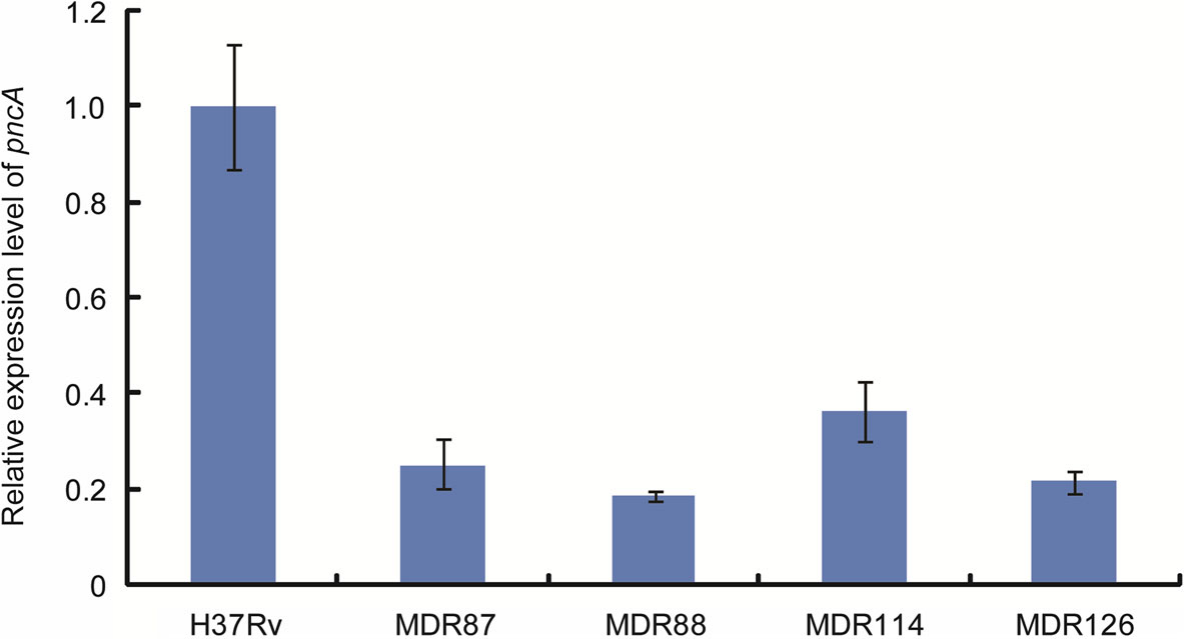
Relative expression level of *pncA* gene in 4 *M. tuberculosis* isolates with mutation in the position −11 of promoter region

We further explore whether the substitution at position −11 affected the expression level of *pncA*. As shown in Fig. 1, compared with reference strain H37Rv, the relative expression level of *pncA* in MDR87, MDR88, MDR114 and MDR126 carried the substitution at position −11 were 0.25 fold, 0.19 fold, 0.36 fold and 0.22 fold, respectively. Statistical analysis revealed that the *pncA* expression of strains harboring promoter mutation at position −11 was significantly lower than that of H37Rv (*P* < 0.01).

## Discussion

PZA plays a unique role in the treatment for MDR-TB in both first- and second-line regimens [19, 20]. The prevalence of PZA among MDR-TB thus is a determining factor for initiation of PZA in the therapy regimens for these refractory patients [21]. Here, our data demonstrated that 62.4% of MDR-TB exhibited resistance against PZA in Chongqing, which was similar to a recent literature from Beijing (57.7%) [22], while higher than those from Zhejiang (43.1%) [8], Shanghai (38.5%) [23], United States (38.0%) [19], and Thailand (49.0%) [24]. The high prevalence of PZA resistance among MDR-TB patients from our report indicates that Chongqing is a hotspot of PZA resistance in China. In our study, more than 60% MDR patients received previous anti-TB therapy with PZA, which is significantly higher than the average national level (21.8%) [3]. Hence, we speculate that the high proportion of PZA resistance may be contributed to the high rate of re-treated TB patients. The serious issue on PZA resistance highlights the diminished role of PZA in the treatment for MDR-TB in this setting with high MDR-TB burden. Prior to the use of PZA for treatment of MDR-TB cases, it is essential to perform in vitro susceptibility testing against PZA to formulate a suitable regimen [21].

Another important finding from our observation was that we observed that there were high correlation between PZA resistance and several other drugs’ resistance, including OFLX, second-line injectable drugs, and PAS. Similar to our findings, a recent report from Alame-Emane and colleagues has revealed that PZA resistance in *M. tuberculosis* arises after RIF and fluoroquinolone (FQ) resistance [25]. Genetic mutations constitute the most important mechanism conferring drug resistance in *M. tuberculosis* [20]. Exposure to bacterial species to antimicrobial agents, including RIF, FQ and the aminoglycosides, induces the production of oxygen radicals, thereby conferring high frequency mutagenesis [25–27]. Considering long duration of anti-TB treatment, we hypothesize that MDR bacteria will harbor more genetic mutations induced by prolonged exposure to these drugs, which may be responsible for the potential cross resistance between PZA and other drugs in our study.

In vitro susceptibility against PZA is essential for proper management of MDR-TB with regimen containing PZA [21]. However, phenotypic DST for PZA is not routinely performed due to the requirement of harshly acidic environment [17]. Molecular method based on detecting the mutations in *pncA* and *rpsA* serves as an alternative to predict the PZA susceptibility in *M. tuberculosis* [9]. In this study, our data demonstrated that genetic alternations in *pncA* confer 88.0% of PZA resistance among MDR-TB in Chongqing. A number of studies have demonstrated a diverse prevalence of *pncA* mutation among PZA resistant isolates in different regions, ranging from 45.7% in Brazil [28], 70.6% in Iran [29], 75.0% in Thailand [24], 78.0% in Zhejiang [8], 84.6% in Southern China [9], and 94.1% in Sweden [30]. Hence, *pncA* mutations may differ from one geographic region to another. In addition, we found that *pncA* mutations exhibited great diversity, and the most frequent mutant type in codon 142 only accounted for approximate 12% of PZA resistant isolates, which was also different from reports from other regions [8, 9]. Given the diversity of *pncA* mutations within more than 500-bp long segment, DNA sequencing of the entire *pncA* is more effective for verification of PZA resistance rather than the routine methods by covering the mutant hotspots.

In addition, the third frequent mutation identified in this study was located at position −11 of the *pncA* promoter region. In line with our observation, numerous literatures have observed this mutant type in PZA-resistant *M. tuberculosis* isolates [31, 32]. We found that this substitution at position −11 was associated with low level of *pncA* expression, which was also consistent to the observation from Sheen et al. [31]. The anti-TB activity of PZA depends on the transformation to POA by PZAse, which is encoded by *pncA* gene. The loss of *pncA* transcriptional level may result in the relative low PZAse activity, which is further associated with the phenotypic PZA resistance. Our results suggest that the promoter region of *pncA* is recommended to be included in the sequence analysis of *pncA* gene.

We acknowledge several limitations of this study. First, the small sample size is a major limitation of our report. And all the strains collected from one region also reduce their representativeness and thereby constrict the generalizability of findings. Further wider sampling will give more credence to this study. Second, although the primary data of this study suggest that loss of *pncA* expression caused by promoter mutation confers PZA resistance in MDR-TB isolates, we could give no biochemical or transgenic substantiation of this statement. Therefore, there is an urgent need to confirm our findings with more experimental evidences in the future. Third, the high diversity of *pncA* mutations in MTB-TB isolates from Chongqing underscores previous findings that there is no clear hotspot for *pncA* mutations [30], and several novel mutations in *pncA* gene were found for first time among PZA-resistant isolates. Despite being highly correlated with the loss of PZA susceptibility, further experiments will be carried out to clarify the potential contributions of these mutations to PZA resistance. Nevertheless, our report firstly described the molecular characteristics of PZA resistance among MDR-TB isolates from Southern China, which provides important hints to diagnose PZA resistance and help guide therapy with PZA for MDR-TB patients in this region with high MDR-TB burden.

## Conclusion

In conclusion, our data have demonstrated that the analysis of the *pncA* gene rather than *rpsA* gene provides rapid and accurate information regarding PZA susceptibility for MDR-TB isolates in Chongqing. In addition, loss of *pncA* expression caused by promoter mutation confers PZA resistance in MDR-TB isolates. Considering the high degree of diversity of *pncA* gene mutations, DNA sequencing of the entire *pncA* is more effective for verification of PZA resistance rather than the routine methods by covering the mutant hotspots. The high prevalence of PZA resistance among MDR highlights the diminished role of PZA in the treatment for MDR-TB in this setting with high TB burden.

## Abbreviations

AMK: Amikacin
CAP: Capromycin
CIs: Confidence intervals
DST: Drug susceptibility testing
EMB: Ethambutol
INH: Isoniazid
KAN: Kanamycin
MDR-TB: Multidrug-resistant tuberculosis
OFLX: Ofloxacin
ORs: Odds ratios
PAS: P-aminosalicylic acid
POA: Pyrazinoci acid
PTO: Protionamide
PZA: Pyrazinamide
PZase: Pyrazinamidase
RIF: Rifampicin
SM: Streptomycin

## References

[1] Gandhi NR, Nunn P, Dheda K, Schaaf HS, Zignol M, van Soolingen D, Jensen P, Bayona J (2010). Multidrug-resistant and extensively drug-resistant tuberculosis: a threat to global control of tuberculosis. Lancet.

[2] Zhang Z, Pang Y, Wang Y, Liu C, Zhao Y (2014). Beijing genotype of Mycobacterium tuberculosis is significantly associated with linezolid resistance in multidrug-resistant and extensively drug-resistant tuberculosis in China. Int J Antimicrob Agents.

[3] Zhao Y, Xu S, Wang L, Chin DP, Wang S, Jiang G, Xia H, Zhou Y, Li Q, Ou X (2012). National survey of drug-resistant tuberculosis in China. N Engl J Med.

[4] Orenstein EW, Basu S, Shah NS, Andrews JR, Friedland GH, Moll AP, Gandhi NR, Galvani AP (2009). Treatment outcomes among patients with multidrug-resistant tuberculosis: systematic review and meta-analysis. Lancet Infect Dis.

[5] Verdugo D, Fallows D, Ahuja S, Schluger N, Kreiswirth B, Mathema B (2015). Epidemiologic Correlates of Pyrazinamide-Resistant Mycobacterium tuberculosis in New York City. Antimicrob Agents Chemother.

[6] Zhang Y, Mitchison D (2003). The curious characteristics of pyrazinamide: a review. Int J Tuberc Lung Dis.

[7] Scorpio A, Zhang Y (1996). Mutations in pncA, a gene encoding pyrazinamidase/nicotinamidase, cause resistance to the antituberculous drug pyrazinamide in tubercle bacillus. Nat Med.

[8] Xia Q, Zhao LL, Li F, Fan YM, Chen YY, Wu BB, Liu ZW, Pan AZ, Zhu M (2015). Phenotypic and genotypic characterization of pyrazinamide resistance among multidrug-resistant Mycobacterium tuberculosis isolates in Zhejiang. China Antimicrob Agents Chemother.

[9] Tan Y, Hu Z, Zhang T, Cai X, Kuang H, Liu Y, Chen J, Yang F, Zhang K, Tan S (2014). Role of pncA and rpsA gene sequencing in detection of pyrazinamide resistance in Mycobacterium tuberculosis isolates from southern China. J Clin Microbiol.

[10] Shi W, Zhang X, Jiang X, Yuan H, Lee JS, Barry CE, Wang H, Zhang W, Zhang Y (2011). Pyrazinamide inhibits trans-translation in Mycobacterium tuberculosis. Science.

[11] Zhang D, An J, Wang J, Hu C, Wang Z, Zhang R, Wang Y, Pang Y (2013). Molecular typing and drug susceptibility of Mycobacterium tuberculosis isolates from Chongqing Municipality, China. Infect Genet Evol.

[12] Zhang D, An J, Wang Y, Pang Y (2014). Genetic diversity of multidrug-resistant tuberculosis in a resource-limited region of China. Int J Infect Dis.

[13] World Health Organization: Global tuberculosis report 2015. Geneva: World Health Organization; 2015.

[14] World Health Organization. Anti-tuberculosis drug resistance in the world. Geneva: World Health Organization; 2008.

[15] Pang Y, Zhou Y, Zhao B, Liu G, Jiang G, Xia H, Song Y, Shang Y, Wang S, Zhao YL (2012). Spoligotyping and drug resistance analysis of Mycobacterium tuberculosis strains from national survey in China. PLoS One.

[16] Zhang Z, Lu J, Wang Y, Pang Y, Zhao Y (2014). Prevalence and molecular characterization of fluoroquinolone-resistant Mycobacterium tuberculosis isolates in China. Antimicrob Agents Chemother.

[17] Pang Y, Wang Z, Zheng H, Song Y, Wang Y, Zhao Y (2015). Pyrazinamide resistance determined by liquid culture at low pH better correlates with genetic mutations in MDR tuberculosis isolates. J Microbiol Methods.

[18] Pang Y, Lu J, Wang Y, Song Y, Wang S, Zhao Y (2013). Study of the rifampin monoresistance mechanism in Mycobacterium tuberculosis. Antimicrob Agents Chemother.

[19] Kurbatova EV, Cavanaugh JS, Dalton T, Click ES, Cegielski JP (2013). Epidemiology of pyrazinamide-resistant tuberculosis in the United States, 1999-2009. Clin Infect Dis.

[20] Zhang Y, Yew WW (2015). Mechanisms of drug resistance in Mycobacterium tuberculosis: update 2015. Int J Tuberc Lung Dis.

[21] Zhang Y, Chiu Chang K, Leung CC, Wai Yew W, Gicquel B, Fallows D, Kaplan G, Chaisson RE, Zhang W (2012). ‘Z(S)-MDR-TB’ versus ‘Z(R)-MDR-TB’: improving treatment of MDR-TB by identifying pyrazinamide susceptibility. Emerg Microbes Infect.

[22] Gu Y, Yu X, Jiang G, Wang X, Ma Y, Li Y, Huang H (2016). Pyrazinamide resistance among multidrug-resistant tuberculosis clinical isolates in a national referral center of China and its correlations with pncA, rpsA, and panD gene mutations. Diagn Microbiol Infect Dis.

[23] Xu P, Wu J, Yang C, Luo T, Shen X, Zhang Y, Nsofor CA, Zhu G, Gicquel B, Gao Q (2016). Prevalence and transmission of pyrazinamide resistant Mycobacterium tuberculosis in China. Tuberculosis (Edinb).

[24] Jonmalung J, Prammananan T, Leechawengwongs M, Chaiprasert A (2010). Surveillance of pyrazinamide susceptibility among multidrug-resistant Mycobacterium tuberculosis isolates from Siriraj Hospital. Thailand BMC Microbiol.

[25] Alame-Emane AK, Xu P, Pierre-Audigier C, Cadet-Daniel V, Shen X, Sraouia M, Siawaya JF, Takiff H, Gao Q, Gicquel B (2015). Pyrazinamide resistance in Mycobacterium tuberculosis arises after rifampicin and fluoroquinolone resistance. Int J Tuberc Lung Dis.

[26] Ysern P, Clerch B, Castano M, Gibert I, Barbe J, Llagostera M (1990). Induction of SOS genes in Escherichia coli and mutagenesis in Salmonella typhimurium by fluoroquinolones. Mutagenesis.

[27] Baharoglu Z, Mazel D (2011). Vibrio cholerae triggers SOS and mutagenesis in response to a wide range of antibiotics: a route towards multiresistance. Antimicrob Agents Chemother.

[28] Bhuju S, Fonseca Lde S, Marsico AG, de Oliveira Vieira GB, Sobral LF, Stehr M, Singh M, Saad MH (2013). Mycobacterium tuberculosis isolates from Rio de Janeiro reveal unusually low correlation between pyrazinamide resistance and mutations in the pncA gene. Infect Genet Evol.

[29] Doustdar F, Khosravi AD, Farnia P (2009). Mycobacterium tuberculosis genotypic diversity in pyrazinamide-resistant isolates of Iran. Microb Drug Resist.

[30] Jureen P, Werngren J, Toro JC, Hoffner S (2008). Pyrazinamide resistance and pncA gene mutations in Mycobacterium tuberculosis. Antimicrob Agents Chemother.

[31] Sheen P, Lozano K, Gilman RH, Valencia HJ, Loli S, Fuentes P, Grandjean L, Zimic M (2013). pncA gene expression and prediction factors on pyrazinamide resistance in Mycobacterium tuberculosis. Tuberculosis (Edinb).

[32] Portugal I, Barreiro L, Moniz-Pereira J, Brum L (2004). pncA mutations in pyrazinamide-resistant Mycobacterium tuberculosis isolates in Portugal. Antimicrob Agents Chemother.

